# Antibiotics, obesity and the link to microbes - what are we doing to our children?

**DOI:** 10.1186/s12916-016-0605-7

**Published:** 2016-04-19

**Authors:** Olli Turta, Samuli Rautava

**Affiliations:** Department of Paediatrics, University of Turku and Turku University Hospital, Turku, Finland

**Keywords:** Antibiotics, Gut microbiota, Infant, Neonate, Obesity, Overweight

## Abstract

**Background:**

Childhood obesity and overweight are among the greatest health challenges in the pediatric population. Obese individuals exhibit marked differences in the composition of the intestinal microbial community as compared to lean subjects. These changes in the gut microbiota precede the clinical manifestation of overweight. Convincing experimental data suggest a causal role for intestinal microbes in the development of obesity and associated metabolic disorders.

**Discussion:**

Exposure to antibiotics exerts a devastating impact on the intestinal microbial community. Epidemiological studies have provided evidence indicating that early or repeated childhood exposure to antibiotics is associated with increased risk of overweight later in childhood but the causal role of this exposure in obesity development is not clear. However, data from studies conducted using experimental animal models indicate that antibiotic-induced changes in the gut microbiota influence host metabolism and lead to fat accumulation. The intestinal microbiota perturbation caused by antibiotic exposure in the perinatal period appears to program the host to an obesity-prone metabolic phenotype, which persists after the antibiotics have been discontinued and the gut microbiota has recovered. These observations may have serious implications in the clinical setting, since a substantial number of human infants are subjected to antibiotic treatment through the mother during delivery or directly in the immediate neonatal period. The clinical significance of these exposures remains unknown.

**Summary:**

Prudent use of antibiotics is paramount not only to reduce the propagation of antibiotic-resistant organisms but also to minimize the potentially detrimental long-term metabolic consequences of early antibiotic exposure. Improved means of reliably detecting neonates with bacterial infection would reduce the need for empirical antibiotic exposure initiated based on nonspecific symptoms and signs or risk factors. Finally, means to support healthy microbial contact in neonates and infants requiring antibiotic treatment are needed.

## Background

The increasing prevalence of childhood obesity is one of the greatest challenges facing medical professionals caring for infants and children. According to a recent report from the United States, more than one in six children and youths between the ages of two and 19 years are obese and more than one in three are overweight [1]. Overweight and obese children are at a high risk of becoming obese adults [2]. The detrimental health consequences of obesity – increased risk of metabolic and cardiovascular disease, musculoskeletal problems as well as psychosocial issues – may manifest already in childhood and become more prevalent with increasing age. Effective strategies for reducing the risk of obesity are desperately needed. Devising such means is dependent on an improved understanding of the factors involved in obesity development. Recent data suggest that the developmental pathway leading to obesity may begin already in infancy and that childhood overweight is a strong predictor of later obesity [3]. Early life programming towards a phenotype prone to obesity may occur as a result of intrauterine or extrauterine influences [3], some of which may be amenable to intervention. As discussed in detail below, reducing antibiotic exposure in the perinatal period or in early infancy, which has recently been associated with an increased risk of obesity development, may be one means of combatting the obesity epidemic.

Overweight and obesity are highly hereditary because of both genetic predisposition and learned behavioral traits. However, hereditary factors fail to explain the massive increase in the prevalence of obesity in populations across the globe. The simple proximal explanation for obesity is excessive energy intake in comparison to energy expenditure. The obesity epidemic may accordingly be explained by changes related to lifestyle which have occurred during the past decades - our increasingly sedentary way of life combined with the all-to-common Western diet high in fat, carbohydrates and energy but low in fiber. Recent advances in scientific research have revealed intriguing mediating factors between the diet, host energy metabolism and the obese phenotype. Namely, the indigenous intestinal microbiota has been suggested to not only be influenced by diet but also to play a causal role in the development of obesity (reviewed in [4]).

The association between the composition of the intestinal microbiota and obesity has been demonstrated by studies showing differences in gut microbiota composition between obese and lean humans [5, 6] and more so in experimental animals. Obesity is associated with an increased abundance of the phylum Firmucutes and a decrease in Bacteroidetes [5]. These gut microbiota alterations are at least partially attributable to diet, since studies conducted using experimental models have demonstrated that animals fed a western-type diet not only display excessive weight gain but exhibit an increase in intestinal Firmicutes and a decrease in Bacteroidetes resembling the changes seen in obese humans [7]. Conversely, weight loss resulting from a low-energy diet is accompanied by a shift in gut microbiota composition to resemble that of lean individuals with a decrease in Firmicutes and an increase in Bacteroidetes [5]. Consuming a Western-type diet is associated with reduced gut microbiota diversity and higher abundance of potential pathogens in both experimental animals and human subjects [8, 9]. These diet-induced changes in gut microbiota are most likely caused by altered availability of substrates for different bacteria, but there are also data indicating that an average Western diet contains fewer viable microbes than a diet that is in accordance with current recommendations [10]. These data conjointly demonstrate an intimate association between the diet, obesity and the gut microbiota. Interestingly, there also appears to be a causal network linking intestinal microbes to the development of obesity.

The direct causal role of intestinal microbes in the development of overweight and obesity has been demonstrated in experimental studies in which the obese phenotype may be transferred to germ-free animals merely by colonizing them with the gut microbiota from obese animals [7] or humans [11]. The mechanisms by which gut microbes may induce weight accumulation have also begun to unravel. The intestinal microbiota associated with obesity has been shown to increase intestinal energy uptake from the diet [12] and its storage in the host [13]. Furthermore, gut microbes appear to modulate the expression of several host genes associated with metabolism at least in part *via* microbial metabolites including short chain fatty acids [reviewed in 4], the production of which is influenced by dietary substrates. The low-grade inflammation characteristic of obesity and metabolic disease may also be induced and propagated by interaction with certain intestinal microbes [4]. It is also of note that prospective clinical studies have shown that changes in gut microbiota composition precede the development of overweight and obesity and may be observed already in early infancy [14–16]. In particular, a high abundance of intestinal bifidobacteria in early life appears to be associated with lower risk of overweight [15, 16], whereas high amounts of *Bacteroides fragilis* increase the risk of obesity development [14]. It is therefore likely that factors that exert an impact on gut microbiota composition in early life may also modulate the risk of obesity development. As alluded to above, a diet rich in energy and fat is known to divert the intestinal microbial population toward a composition, which may promote weight gain [7]. Conversely, a diet rich in fermentable but non-digestible carbohydrates has been shown to alleviate obesity in children *via* modulation of the gut microbiota [17]. The purpose of this paper is to discuss the potentially detrimental effects of non-dietary factors and particularly exposure to antibiotics, which may disrupt the gut microbiota in early life and consequently increase the risk of obesity in children.

## Discussion

Antimicrobial agents are among the most frequently used pharmaceuticals in infants and children. Cox and Blaser have recently estimated, that by two years of age, children in the United States have on average received nearly three courses of antibiotics and the number of courses increases to approximately ten by the age of ten years [18]. It is likely that these figures are relatively similar for other developed countries, albeit considerable differences in the frequency of antibiotic use have been reported between countries and regions. The use of antibiotics is particularly prevalent in the perinatal period. Intrapartum antibiotic prophylaxis is administered to the mother to prevent preterm birth and to reduce the risk of maternal and neonatal infections. According to recent publications [19, 20], 33–39 % of newborn infants are exposed to antibiotics through the mother during delivery. In the immediate neonatal period, empirical antibiotic use is also frequent because of the high risk of invasive bacterial infections in the neonate and the difficulty of accurately identifying newborn infants with septicemia. According to current recommendations [21], all symptomatic neonates and certain asymptomatic individuals with a high risk of bacterial infection should receive empirical antibiotic therapy. Consequently, more than 5 % of neonates have been reported to receive antibiotics [20] even though the incidence of culture-proven sepsis in newborn infants is less than one in one thousand neonates [22]. The well-known untoward consequences of antibiotic exposure include the propagation of microbial antibiotic resistance and financial cost. We are only beginning to understand the potentially detrimental long-term impact of early antibiotic exposure on the intestinal microbiota and child health.

Given the mechanism of action of antimicrobial agents, it is hardly surprising that antibiotic therapy inflicts a profound impact on intestinal microbial ecology [23, 24]. In adults, the gut microbiota is thought to be restored to its steady state relatively rapidly after initial perturbation when antibiotic exposure is discontinued, but antibiotic-associated diarrhea, particularly that caused by *Clostridium difficile*, is a relatively common complication of treatment. Nonetheless, there are data suggesting that more prolonged gut microbiota alterations may result from repeated antibiotic exposure [24] and even a single seven-day course of clindamycin has been reported to induce gut microbiota changes detectable two years after exposure in healthy volunteers [25]. Considerably less is known about the impact of antibiotic exposure on the developing intestinal microbiota in infants and young children.

Antibiotic exposure in the neonatal period has a considerable impact on early gut microbiota development. In a study of nine term neonates subjected to treatment with ampicillin and gentamicin, this antibiotic exposure was associated with an increase in fecal Proteobacteria and a decrease in Actinobacteria and particularly *Bifidobacterium* species at four weeks of age as compared to non-exposed neonates [26]. The higher number of fecal Proteobacteria in antibiotic-exposed infants persisted until the end of the follow-up at eight weeks of age while the abundance of Actinobacteria had recovered by that time point. Similar results have been reported by Arboleya and co-workers [27], who found that both intrapartum and neonatal antibiotic exposure increased the abundance of fecal Enterobacteriacaea during the first three months of life. It is of note that the impact of maternal intrapartum antibiotic administration on the breast milk microbiota or the significance of neonatal antibiotic exposure *via* breast milk are currently not known. In preterm infants, empirical antibiotic therapy based on a clinical suspicion of infection in the first week of life resulted in a higher relative abundance of *Enterobacterium* species and lower gut microbiota diversity at the age of three weeks [28]. The clinical significance of the overrepresentation of Proteobacteria and *Enterobacterium* species in particular is not known. Nonetheless, there are compelling data indicating that early life antibiotic exposure markedly increases the risk of overweight and obesity in later life.

A number of well-conducted epidemiological studies suggest that antibiotic exposure in infancy is associated with a higher body mass index (BMI) [29, 30] as well as an increased risk of overweight [31, 32] and obesity [33] later in childhood. A close reading of these reports reveals that exposure to antibiotics appears to be particularly detrimental during the first six months of life [29–31]. In an epidemiological study of more than 11,000 children from the United Kingdom, antibiotic exposure during this time period significantly increased the risk of overweight at 38 months of age (OR 1.22 after adjusting for potential confounding factors; *p* = 0.029). Interestingly, boys may be more affected by early antibiotic exposure than girls [31, 32]. Antibiotic exposure during the first six months of life was associated with overweight at the age of two years in boys (adjusted OR 1.34 with 95 % CI 1.06–1.66) in a cohort study of 12,000 children from Finland [31] but the association was not detected in girls (adjusted OR 1.16 with 95 % CI 0.87–1.56). Furthermore, a dose-response relationship pertaining to the number of courses of antibiotics during the first two years of life and the risk of childhood obesity was observed in a cohort of more than 64,000 children from the United States [33]. These epidemiological studies based on tens of thousands of subjects clearly demonstrate an association between early antibiotic exposure and the development of overweight. However, establishing a causal link between the two is more difficult.

A potential confounding factor in the reports indicating an association between early antibiotic use and subsequent overweight or obesity are the infections because of which the antibiotic therapy was initiated. The large epidemiological studies published thus far have based their estimates of antibiotic exposure either on prescription records [31–33] or parental questionnaires [30] or interviews [29]. It is reasonable to assume that most of the antibiotic courses in these studies have been targeted against infections of the respiratory tract, ears, skin and urinary tract. Infectious disease in early childhood has been suggested to play both a causative and protective role in the development of chronic diseases such as asthma and allergic disease and type I diabetes mellitus [34]. Interestingly, childhood chronic diseases such as asthma [35] and inflammatory bowel disease [36] have also been linked to antibiotic exposure in infancy but it is not clear whether the antibiotics or the infections for which they have been prescribed play a causal role in their development. A recent report suggests – contrary to what has previously been thought - that infectious disease *per se* and not antibiotic use increases the risk of psoriasis in the pediatric population [37]. Similar causal patterns may also be involved in the development of obesity since aberrant immune activation and a low-grade inflammatory state are hallmarks of obesity and metabolic disease. It is therefore possible that antibiotic use is merely a marker for infectious or immunomodulatory events playing a role in the pathogenetic pathway leading to weight accumulation. Investigating the impact of antibiotic exposure on the development of disease in infants or children not suffering from infectious disease in randomized clinical trials is not possible for obvious ethical reasons. Evidence of antibiotic exposure in the absence of infection is thus needed from both epidemiological studies and experimental animal models to establish a causal connection between antibiotic exposure and the development of obesity.

A substantial number of subjects are exposed to antimicrobial therapy without evidence of infection during the perinatal period [20]. The wide-spread – and often well-justified - practices of prophylactic or empirical antibiotic use during delivery and in the immediate neonatal period offer a unique opportunity to study the impact of early antibiotic exposure without the confounding effect of concomitant infection. Large-scale studies with clinical outcome measures using such an approach have to our knowledge not been published and it is likely that the data acquisition methods in the epidemiological studies discussed above [29–33] are not sensitive to antibiotics administered immediately before and after birth. Unpublished results from our laboratory suggest that antibiotic use both during delivery and in the neonatal period in infants not suffering from infections results in perturbations in intestinal microecology during the first six months of life. However, the clinical sequelae of this aberrant gut colonization are not known.

It is well-established that other disturbances in perinatal microbial contact may increase the risk of obesity. Infants born by caesarean section (CS) delivery do not acquire the physiological inoculum of colonizing microbes from the birth canal and maternal gut that vaginally born infants receive. Substantial differences in gut microbiota between infants born by CS or through the vaginal route have been reported in early infancy [38]. In the immediate neonatal period, vaginally delivered newborns are colonized by maternal vaginal lactobacilli whereas maternal skin microbes are detected in the feces of individuals born by CS [38]. The differences in gut microbiota composition between vaginally and CS delivered children extend until the age of seven years [39]. The clinical significance of this phenomenon is demonstrated by a recent systematic review and meta-analysis of epidemiological studies indicating that the risk of childhood obesity in infants born by CS is 1.34-fold (95 % CI 1.18–1.51) as compared to vaginally-born subjects even if maternal BMI is taken into consideration as a confounding factor [40]. It should be borne in mind that in most centers prophylactic antibiotics are administered to mothers undergoing CS. According a Cochrane systematic review, antibiotic use before elective CS reduces infectious complications in the mother but its effects on the child are unknown [41]. It is possible that at least a part of the reported association between CS delivery and the risk of obesity may in fact be mediated by antibiotic exposure. In support of this notion, antibiotic use during pregnancy and CS delivery appear to have an additive effect on BMI in childhood [42].

The strongest corroboration for the hypothesis of early antibiotic exposure causally increasing the risk of later-life obesity stems from experimental studies conducted using animal models. A series of experiments conducted in Dr. Blaser’s laboratory at the New York School of Medicine has demonstrated that exposure to low-dose antibiotics from weaning results in increased fat-mass accumulation in mice [43]. This increase in adiposity appears to be mediated by antibiotic-induced changes in gut microbiota composition leading to increased short-chain fatty acid production and altered host metabolism including upregulation of genes involved in lipogenesis. The observations according to which antibiotic exposure at a subtherapeutic dose may increase weight accumulation are highly relevant given the fact that production animals are fed antibiotics in order to increase weight gain in many countries and, as a result, human subjects may be exposed to low-dose antibiotics by consuming meat products.

A subsequent report from the same laboratory has further increased our understanding of these phenomena [44]. Maternal exposure to low-dose penicillin during late gestation and lactation had a more pronounced impact on weight and fat accumulation than direct exposure after weaning. These data imply that there is a critical time window in which antibiotic exposure may be particularly detrimental. Male mice were more profoundly affected than females by perinatal antibiotic exposure, which is consistent with the epidemiological data showing increased risk of overweight and obesity in antibiotic-exposed boys discussed above. The fact that the obese phenotype observed after antibiotic exposure is dependent on the gut microbiota is demonstrated by experiments showing that fat accumulation may be induced in non-exposed germ-free animals by colonizing them with the altered gut microbiota from antibiotic-exposed mice. Finally, and perhaps most alarmingly, it appears that early antibiotic exposure may permanently program host metabolism to an obese phenotype, since the animals remained fat after the antibiotic exposure was discontinued even when the gut microbiota recovered to resemble that of normal-weight mice.

## Summary

The association and possible causal relationship between early-life antibiotic exposure and the development of overweight and obesity raise concerns about current medical practices but may also provide opportunities to combat the obesity epidemic. It is clear that antibiotics are often used for the treatment of conditions, which are known to primarily be of viral origin or in instances where there is little or no evidence of efficacy. Prudent use of antibiotics is paramount to limit the propagation of antimicrobial resistance in pathogens. Fortunately, there are data indicating that antibiotic use in children has reduced during recent years [45]. Research assessing the safety and efficacy of antimicrobial therapy should address the long-term impact of the interventions. In particular, prophylactic antibiotic use in the perinatal period should be subjected to rigorous scrutiny.

According to current recommendations, newborn infants are to be treated with empirical antibiotic therapy based on non-specific symptoms and signs of infection or if the risk of infection has been estimated high based on factors such as maternal chorionamnionitis or colonization with group B *Streptococcus* [21]. These practices are necessary even though they result in antibiotic exposure in a large number of non-infected neonates, because of lack of specific and sensitive markers to detect infection early in the course of disease. Developing such means would result in a significant reduction of antibiotic use during the first days of life and, given the potentially unfavorable long-term effects of early antibiotic exposure, might entail a considerable health impact.

Antibiotic therapy is often a necessary and life-saving intervention in infants and children suffering from bacterial infection. Even with improved diagnostic methods and prudence in assessing the need for antibiotics, a considerable number of subjects need to be exposed to these drugs. To minimize the long-term risks involved with early antibiotic exposure, adjunct therapies aiming to alleviate its detrimental impact on the gut microbiota and consequently host metabolism should be developed. Oral bacteriotherapy with probiotic organisms might offer a means of modulating both the intestinal microbial community and host physiology. The efficacy of probiotics is well-established in the prevention of antibiotic-associated diarrhea [46]. Future research will reveal if the promise of probiotics holds true for the risk of obesity.
